# Long-Term Neuromodulatory Effects of Repetitive Transcranial Magnetic Stimulation (rTMS) on Plasmatic Matrix Metalloproteinases (MMPs) Levels and Visuospatial Abilities in Mild Cognitive Impairment (MCI)

**DOI:** 10.3390/ijms24043231

**Published:** 2023-02-06

**Authors:** Giovanni Cirillo, Roberta Pepe, Mattia Siciliano, Domenico Ippolito, Dario Ricciardi, Manuela de Stefano, Daniela Buonanno, Danilo Atripaldi, Salvatore Abbadessa, Brunella Perfetto, Minoo Sharbafshaaer, Giovanna Sepe, Simona Bonavita, Alessandro Iavarone, Vincenzo Todisco, Michele Papa, Gioacchino Tedeschi, Sabrina Esposito, Francesca Trojsi

**Affiliations:** 1 Neuronal Networks Morphology & Systems Biology Lab, Division of Human Anatomy, Department of Mental and Physical Health and Preventive Medicine, University of Campania “Luigi Vanvitelli”, 80138 Naples, Italy; 2 First Division of Neurology, Department of Advanced Medical and Surgical Sciences, University of Campania “Luigi Vanvitelli”, 80138 Naples, Italy; 3 Neurologic Unit, Centro Traumatologico Ortopedico (CTO) Hospital, Azienda Ospedaliera di Rilievo Nazionale (AORN) “Ospedali Dei Colli”, 80138 Naples, Italy; 4 Department of Precision Medicine, University of Campania “Luigi Vanvitelli”, 80138 Naples, Italy; 5 Department of Experimental Medicine, University of Campania “Luigi Vanvitelli”, 80138 Naples, Italy

**Keywords:** mild cognitive impairment, metalloprotease, neuromodulation, neuroinflammation

## Abstract

Repetitive transcranial magnetic stimulation (rTMS) is a non-invasive neuromodulation technique that is used against cognitive impairment in mild cognitive impairment (MCI) and Alzheimer’s disease (AD). However, the neurobiological mechanisms underlying the rTMS therapeutic effects are still only partially investigated. Maladaptive plasticity, glial activation, and neuroinflammation, including metalloproteases (MMPs) activation, might represent new potential targets of the neurodegenerative process and progression from MCI to AD. In this study, we aimed to evaluate the effects of bilateral rTMS over the dorsolateral prefrontal cortex (DLPFC) on plasmatic levels of MMP1, -2, -9, and -10; MMPs-related tissue inhibitors TIMP1 and TIMP2; and cognitive performances in MCI patients. Patients received high-frequency (10 Hz) rTMS (MCI-TMS, *n* = 9) or sham stimulation (MCI-C, *n* = 9) daily for four weeks, and they were monitored for six months after TMS. The plasmatic levels of MMPs and TIMPs and the cognitive and behavioral scores, based on the Repeatable Battery for the Assessment of Neuropsychological Status (RBANS), Beck Depression Inventory II, Beck Anxiety Inventory, and Apathy Evaluation Scale, were assessed at baseline (T0) and after 1 month (T1) and 6 months (T2) since rTMS. In the MCI-TMS group, at T2, plasmatic levels of MMP1, -9, and -10 were reduced and paralleled by increased plasmatic levels of TIMP1 and TIMP2 and improvement of visuospatial performances. In conclusion, our findings suggest that targeting DLPFC by rTMS might result in the long-term modulation of the MMPs/TIMPs system in MCI patients and the neurobiological mechanisms associated with MCI progression to dementia.

## 1. Introduction

Mild cognitive impairment (MCI) is a neurological disorder occurring mainly in older people with cognitive deficits that are not severe enough to warrant a diagnosis of dementia [1]. It has been classified as a prodromal stage of a variety of dementing disorders, including Alzheimer’s disease (AD) [2]. Cognitive impairment associated with MCI can affect all cognitive domains, including memory, language, attention, visuospatial functioning, and executive functions [3].

Pharmacological and non-pharmacological interventions have been proposed and recommended for the treatment of MCI, and among the latter ones, non-invasive brain stimulation (NIBS) techniques, through the modulation of brain circuits [4,5,6,7], have shown clinical efficacy enhancing cognitive performances without significant adverse effects [8,9,10,11]. Among NIBS techniques, the electromagnetic field of the transcranial magnetic stimulation (TMS) penetrates the scalp and the skull, induces an electric field in the brain tissue, and allows a non-invasive activation of the cerebral cortex [12]. In particular, repetitive TMS (rTMS) consists of administrating a series of impulses at the same intensity and frequency targeted to a specific brain area [11], which generates post-stimulation changes affecting action potential and resting membrane potential and then increases synaptic connectivity [4]. In this regard, rTMS has the ability to modulate synaptic plasticity and induce several neural changes at different levels, spanning from the cellular/molecular (i.e., protein phosphorylation, gene activation, and receptor expression) to morpho-functional modifications of neural cells (dendritic spines or axonal/dendritic arborization, neurotransmission, and modulation of glial function) and brain networks [4,9,13,14].

To date, glial cells are active players of synaptic plasticity that are involved in the fine regulation of synaptic transmission and homeostasis and in the metabolic coupling with neurons [15,16]. Astrocytes, in particular, control the homeostasis of neurotrophic factors, maintaining the balance between their secretion and their degradation, mainly occurring through the system of metalloproteases (MMPs) [17,18,19,20,21]. MMPs are a family of endopeptidases; they are secreted and expressed by neurons and glia and contribute to synaptic plasticity by acting on the remodeling and growth of neuronal elements through their proteolytic action on extra-cellular matrix (ECM) proteins [22,23,24,25,26]. These molecules may be secreted into the extracellular space in case of brain damage or during neuroinflammatory/neurodegenerative process by the activated microglia and astrocytes [27,28,29], a process called reactive gliosis and characterized by the release of pro-inflammatory molecules and secretion of neurotrophins [30,31]. MMP9 is involved in the cleavage and degradation of the mature nerve growth factor (NGF), thereby inducing the reduction of the brain levels of NGF, possibly contributing to neuronal death [32,33]. Conversely, increased brain levels of the MMP9 inhibitor, such as tissue inhibitors of metalloproteases 1 and 2 (TIMP1 and TIMP2) or blocking MMPs’ activity [23], have been shown to increase NGF expression, reduce reactive gliosis, and restore the synaptic homeostasis [31].

Besides their role in the neurological disorders, MMPs might contribute to the development of cardiovascular diseases and diabetes mellitus, increasing the all-cause mortality [34]: higher plasma levels of MMPs (MMP2 and 10) have been observed in patients with type 1 diabetes [35,36], associated with log-grade inflammation and endothelial dysfunction (MMP2 and MMP9) [37,38] and renal disorders (MMP9) [39]. Altogether, these studies suggest that MMPs might participate in the development of vascular disorders that, in turn, are known to contribute to the establishment of the neurodegenerative process [40,41].

Considering the role of MMPs in (1) the degradation of extracellular proteins, (2) the control of brain neurotrophins’ availability, (3) the activation of neuroinflammatory mechanisms, and (4) brain homeostasis, it is conceivable that MMPs may potentially contribute to the pathogenesis of AD/MCI [42,43]. It has been hypothesized that increased cerebrospinal fluid (CSF) and/or plasma levels of MMPs could be related to the different stages of the neurodegenerative process in AD. Accordingly, a recent study on CSF levels of MMP10 showed a higher risk of progressing to AD in MCI patients with higher CSF levels of this MMP [44]. Moreover, in a chronic neuroinflammatory disease, such as multiple sclerosis, an MMP9 increase was found in patients’ sera and plasma during relapse [45,46,47] and during methylprednisolone treatment [47]. Furthermore, before the appearance of new gadolinium-enhancing lesions, the MMP9/TIMP1 ratio was increased [48], suggesting that the balance between MMP9 and TIMP1 could represent a marker of the permeability of the blood brain–barrier to pro-inflammatory cells and that the neuroinflammatory process is well reflected in the peripheral blood. However, despite this robust evidence in multiple sclerosis, modifications of MMPs’ plasmatic levels at baseline and/or in response to pharmacological or non-pharmacological therapeutic approaches, including NIBS techniques, have not been still explored in AD/MCI.

Based on these considerations, our main objective was to shed light on the short-term and long-term changes of plasmatic levels of some key MMPs (MMP1, -2, -9, and -10) that were found to be related to the neurodegenerative process, as well as their inhibitors (TIMP1 and TIMP2), in a cohort of MCI patients who received bilateral rTMS stimulation of the dorsolateral prefrontal cortex (DLPFC), in comparison to a control group of unstimulated MCI patients. We monitored the neuropsychological performances and the plasmatic levels of both MMPs/TIMPs (1) at baseline, (2) after five weeks, and (3) six months from rTMS stimulation. We aimed at identifying divergent changing patterns of the plasmatic MMPs and TIMPs across time and clinical–molecular correlation in response to the intervention performed.

## 2. Results

### 2.1. Clinical and Neuropsychological Assessment

**Figure 1:**
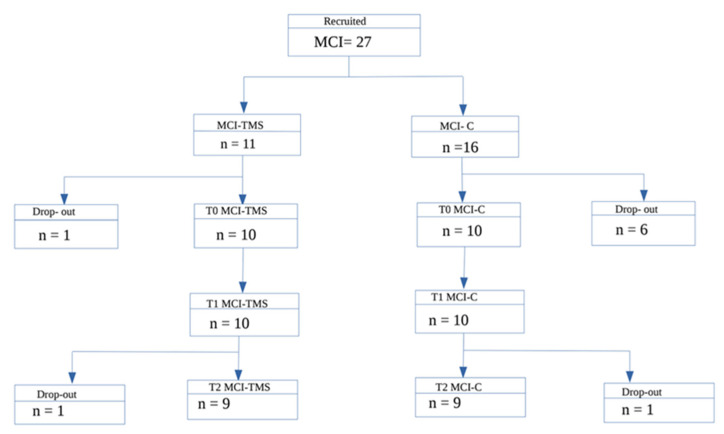
Flowchart of the patients included.

Out of 40 screened subjects, 7 did not fulfill the inclusion criteria for MCI. Among the 33 MCI patients left, 27 signed the informed consent to this study. A total of 11 MCI patients were randomly assigned to the treated group (MCI-TMS), and 16 were assigned to the sham group as controls (MCI-C). However, only 10 patients per group completed the follow-up protocol and were included in the analysis (Figure 1). The demographics and cognitive and behavioral measures at baseline are presented in Table 1. No significant adverse effects (AEs) occurred during the study protocol: we observed transient mild discomfort at the stimulation site (*n* = 3) and mild headache (*n* = 2) in the rTMS group that did not require use of medications. The treatment schedule was completed with a good overall adherence, confirming that TMS is a well-tolerated treatment option, despite being time-consuming.

No significant differences were detected for demographic, clinical, cognitive, and behavioral measures among the groups at baseline (Table 1) and T1 (4 weeks after rTMS) (Table 2). Conversely, neuropsychological assessment at T2 (6 months, 24 weeks later, after rTMS) showed a significant difference in the line orientation test in the group of patients who underwent TMS (Table 3).

### 2.2. rTMS-Induced Modulation of MMPs/TIMPs Plasmatic Levels

**Figure 2:**
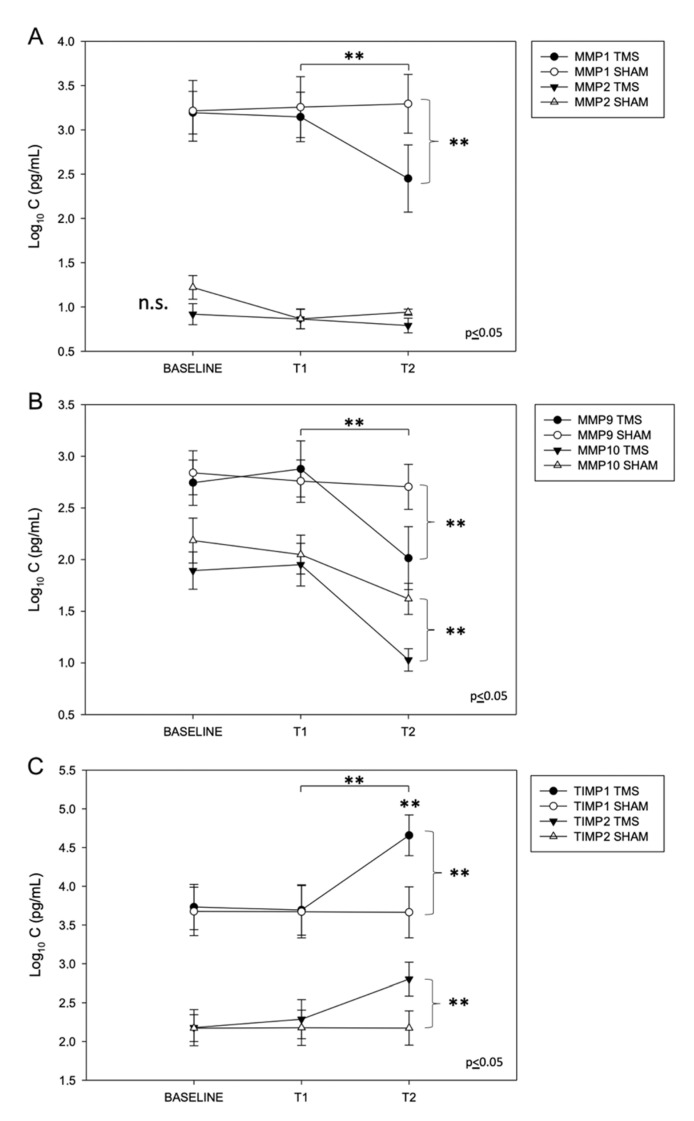
Plasmatic levels of MMPs at baseline and after rTMS. (**A**,**B**) Significant reduction of MMP1, -9, and -10 plasmatic levels 6 months later after rTMS (T2, 24 weeks), compared to the SHAM group and to the levels at baseline and T1 (4 weeks after rTMS). No significant changes were detected for MMP2. (**C**) Significant increase of TIMP1 and TIMP2 plasmatic levels 6 months later after rTMS (T2, 24 weeks), compared to the SHAM group and to the levels at baseline and T1 (4 weeks after rTMS) (data expressed as mean ± standard deviation, ** = *p* ≤ 0.05).

Plasmatic levels of MMP1, -2, -9, and -10 and TIMP1/TIMP2 were evaluated at baseline, after 4 weeks of rTMS (T1), and 6 months after the end of the stimulations (T2, 24 weeks after rTMS). In the stimulated patients, our analysis revealed a significant reduction of the plasmatic levels of MMP1 (2.48 ± 0.9), MMP9 (2.05 ± 0.8), and MMP10 (1.02 ± 0.2) at T2 compared to the sham group (MMP1, 3.35 ± 1.1; MMP9, 2.71 ± 0.6; and MMP10, 1.7 ± 0.3) (** *p* ≤ 0.05) (Figure 2A,B).

No significant changes in the MMP1, -9, and -10 plasmatic levels were detected at T1 compared to the baseline in the two groups (*p* ≥ 0.5). Finally, we did not detect changes of MMP2 levels in the two groups across the experimental conditions.

We further evaluated the plasmatic levels of TIMP1 and TIMP2 at the same time points. We found that both the TIMP1 and TIMP2 levels were increased at T2, six months after rTMS (TIMP1: 4.67 ± 0.8; TIMP2: 2.85 ± 0.4), compared to the MCI-C group (TIMP1, 3.6 ± 0.9; TIMP2, 2.2 ± 0.4) (** *p* ≤ 0.05) (Figure 2C).

No significant changes of TIMP1 and TIMP2 plasmatic levels were detected at T1 compared to the baseline in the two groups (*p* ≥ 0.5). Accordingly, the TIMP1/MMP9 ratio at T2 was significantly increased after rTMS (2.27) compared to the MCI-C group (1.3) (** *p* ≤ 0.05).

## 3. Discussion

Our pilot study revealed for the first time that bilateral rTMS over the DLPFC for 4 weeks was able to significantly modulate at long-term (6 months, 24 weeks later) the plasmatic levels of selected MMPs and TIMPs in MCI patients. In particular, rTMS significantly reduced the plasmatic levels of MMP1, MMP9, and MMP10 and increased the plasmatic levels of their inhibitors, TIMP1 and TIMP2. These biological effects after rTMS, paralleled by improvement of visuospatial perceptive abilities, as revealed by the higher scores on the judgment of line orientation subtest, prompt us to hypothesize that rTMS has a neuroprotective effect.

Impairment of visuospatial abilities has been less investigated in MCI in comparison to disorders of episodic memory, language, and executive functions [49,50,51,52]. Depending on task demands, visuospatial dysfunction has been associated with temporal, parietal, and occipital injuries [53], as well as hemispheric asymmetries in visuospatial processing [54]. Regarding the judgment of the line-orientation test, patients with lesions mainly located in right posterior parietal regions demonstrated a worse performance [54]. However, impairment in judgment of the line-orientation-test performance may also occur in patients with left parietal dysfunction [55]. These findings induced us to hypothesize that the “perceptive” ability of judgment of line orientation is initially processed by the right parietal lobe, with a subsequent detailed categorical spatial analysis mediated by the left parietal region. Additionally, worse performances in regard to the judgment of the line-orientation test have been revealed to be useful for differentiating multi-domain/dysexecutive MCI from non-MCI patients [56]. Based on this background, our findings revealing a long-term improvement of visuospatial abilities after rTMS over the DLPFC bilaterally may be interpreted as a potential effect of rTMS on brain frontoparietal circuits, especially on the so-called “dorsal pathway”, hypothesized to be involved in spatial working memory, visually guided action, and navigation, as well as visuospatial processing [57,58,59], and proven to be impaired in amnestic MCI [51]. However, considering that, in our population, we did not collect structural and/or functional magnetic resonance imaging (MRI) data exploring brain connectivity that would be useful to investigate in vivo the neurobiological effects of neurodegeneration on brain networks, we were not able to verify these effects as a consequence of the rTMS protocol administered to our MCI patients.

Our findings of the reduction of the plasmatic levels of MMP1, MMP9, and MMP10 and of the increase of the plasmatic levels of their inhibitors TIMP1 and TIMP2 as a long-term result after rTMS in MCI patients may reflect potential effect of rTMS on the modulation of neuroinflammatory mechanisms related to neurodegeneration. This interpretation is supported by the fact that MMPs regulate several processes, including inflammation, microglial activation, blood–brain barrier integrity, and apoptosis [60]; they are also pivotal in the neuroinflammatory response to ischemic injury/infection and vascular dementias [27] and AD [61]. In particular, a relationship between the increase of tissue activation of MMP9 and cognitive impairment has been hypothesized in MCI and AD [62], revealing that the expression of MMP9 was upregulated in AD patients in neurofibrillary tangles, neuronal cytoplasm, vascular tissue, and amyloid plaques [63]. On the other hand, the finding of decreased plasma levels of MMPs after six months from rTMS sessions might be hypothesized as a long-term effect of the neuromodulation induced by rTMS treatment on neuroplasticity mechanisms (including availability of neurotrophic factor), taking into account previous evidence of correlations between variable expression/activation of extracellular matrix proteases and the regulation of synaptic plasticity [64]. Among the many MMPs, MMP9 has been most extensively characterized for its essential role in plasticity: this enzyme has a postsynaptic localization [65] with both permissive and instructive roles in plasticity of excitatory synapses [66].

There are several reports regarding the important role of MMP9 in maintaining long-term potentiation (LTP), the major model of synaptic plasticity, both in brain slices and in vivo in different brain structures that were subjected to LTP protocols [66,67,68]. Contrariwise, other evidence showed that LTP was impaired in transgenic rats overexpressing MMP9 in some hippocampal pathways [69]. As for our finding of reduced plasma levels of MMPs in stimulated MCI patients, we can only hypothesize that this result may reflect changes of synaptic plasticity in our MCI-TMS group. This hypothesis may be supported by the current robust evidence of neuromodulation effects of rTMS on synaptic plasticity [4], which is known to be both regulated and reflected by changes of MMPs expression/concentrations in brain tissue and biological fluids [64]. However, the hypothesis of a potential link between synaptic plasticity induced by rTMS and longitudinal changes of plasma levels of MMPs needs to be further addressed in future investigations recurring to the application of advanced neuroimaging techniques for monitoring this association in vivo across time.

Our study has several limitations worth noting, principally consisting in the small sample size and suboptimal statistical design (i.e., analysis of covariance), the lack of neuropsychological and MMPs data in healthy controls, the missing dosages of MMPs levels in cerebrospinal fluid, and the lack of correlation of the neurobiological findings with structural or functional neuroimaging data. Moreover, we did not evaluate the long-term effects of the rTMS in the evolution or transformation to dementia and other potential montages and protocols of stimulation.

## 4. Conclusions

In conclusion, the evidence of our study supports the theory that changes of plasma levels of MMPs may reflect the dynamic neurobiological scenario underlying MCI/AD, characterized by a neuroinflammatory component, which may be modulated by the application of a DLPFC rTMS protocol. Non-invasive brain stimulation by rTMS confirms to be a promising tool for improving cognitive function in MCI patients, offering potential long-term advantages on performances in visuospatial tasks.

Future multimodal analyses using mixed modeling and recurring to both brain stimulation protocols by rTMS and advanced neuroimaging techniques for monitoring neuroplasticity effects of rTMS in vivo together with neuropsychological assessment and dosage of MMPs plasma levels will aid to corroborate the suggested link between clinical and neuroplasticity effects of rTMS and changes of plasma levels of MMPs.

Overall, this study provides pilot data and is a steppingstone to a larger study that could provide more definitive data. Our evidence, as well as the proposed multimodal analyses, will allow us (1) to support the role of brain stimulation protocols by rTMS as valuable tools for cognitive rehabilitation of MCI patients and (2) to validate the potential use of plasma levels of MMPs as biological markers of neuroinflammation and response to treatments inducing neuromodulation.

## 5. Materials and Methods

### 5.1. Study Populations

From January 2018 to February 2020, 40 MCI patients were screened for this study and 20 MCI patients (7 males, 13 females) were recruited from January 2018 to February 2020 at the First Division of Neurology of the University of Campania “Luigi Vanvitelli” (Naples, Italy) and included in the study. Patients were required (1) to meet the “core” criteria for MCI, as defined by the National Institute on Aging-Alzheimer’s Association workgroups on diagnostic guidelines for Alzheimer’s disease [70]; (2) to have a Clinical Dementia Rating (CDR) of 0.5; (3) to have been at an age ≥40 at the onset of cognitive symptoms; and (4) to possess the ability to understand the purpose of the study and to sign the informed consent. The diagnosis of MCI included a formal neuropsychological assessment by Mental Deterioration Battery (MDB) and functional assessment by ADL/IADL.

Exclusion criteria for all subjects were (1) medical illnesses or substance abuse that could interfere with cognitive functioning; (2) any (other) major systemic, psychiatric, or neurological diseases; (3) other causes of brain damage, including lacunae and extensive cerebrovascular disorders at MRI; and (4) contraindications for MRI and TMS, according to the Standard Questionnaire of “The Safety of TMS Consensus Group” [71].

The research was carried out by following the principles expressed in the Declaration of Helsinki. Ethics approval was obtained from the Ethics Committee of the University of Campania “Luigi Vanvitelli” (Prot. N. 241/2017).

### 5.2. Study Design

The study was carried out with a randomized, controlled, double-blind (patient and neuropsychologist) design. According to a previous study carried out by our group [9], patients meeting all the inclusion criteria and none of the exclusion criteria underwent a baseline (T0) neuropsychological examination, using Repeatable Battery for the Assessment of Neuropsychological Status (RBANS) Form A, Beck Depression Inventory II (BDI-II), the Beck Anxiety Inventory (BAI), and the Apathy Evaluation Scale (AES); then they were randomized (http://www.random.org, accessed on 1 May 2019) to the active or sham arm. After the 4-week rTMS (T1), and 6 months after the end of the stimulations (24 weeks, T2), they repeated the neuropsychological assessment. To minimize the learning effect, the RBANS Forms B and C were used in T1 and T2. At the 3 timepoints (T0, T1, and T2), blood samples were collected in all the studied population.

#### 5.2.1. Neuropsychological Assessment

To assess the cognitive functioning of the study groups, we used the RBANS Form A, B, and C [72]. These forms comprise 12 subtests, indexing 5 different cognitive domains (attention, immediate memory, delayed memory, language, and visuospatial/constructional). To assess the behavioral profile of the study groups, we employed Italian versions of the Beck Depression Inventory II (BDI-II), the Beck Anxiety Inventory (BAI) [73], and the Apathy Evaluation Scale (AES) [74]. The BDI-II questionnaire contains 21 self-report items used in clinical and research setting to assess depressive symptoms. The BAI is a questionnaire with 20 self-report items focused on somatic, behavioral, emotional, and cognitive symptoms of anxiety. The AES is an 18-item self-assessment questionnaire that is used for research purposes to evaluate apathy through 4 subscales (cognitive, behavioral, emotional, and other).

#### 5.2.2. TMS Protocol

MCI patients were divided in a double-blind condition into two groups, the active one receiving active TMS (MCI-TMS) and the control sham group (MCI-C). All the patients had no experience of TMS, so they did not know whether they were receiving real or sham TMS. For the MCI-C group, we used a placebo coil, which was identical to the active one and delivered <5% of the magnetic output.

High-frequency (HF, 10 Hz) repetitive TMS (HF-rTMS) was applied over the DLPFC through a figure-of-eight coil (loop diameters of 9 cm) that was mounted on an articulated arm, positioned tangentially to the skull, and connected to a Magstim2 Rapid stimulator (The Magstim Company, Whitland, UK). The stimulation intensity was 80% of the resting motor threshold (RMT), defined as the lowest single pulse intensity required to produce a motor-evoked potential (MEP) greater than 50 μV (peak-to-peak amplitude) on more than five out of ten trials from the contracted contralateral abductor pollicis brevis (APB) [7].

rTMS was applied for ten minutes over the DLPFC (Brodmann areas 8/9) at the point located approximately 5 cm in a parasagittal plane parallel to the point of maximum stimulation of the APB, with the lowest possible intensity in five of ten stimuli [9,75]. Subjects assigned to the MCI-TMS group received over the DLPFC, bilaterally, HF-rTMS (10 Hz) for 10 min (20 trains of stimuli, with each train consisting of 100 pulses and lasting 10 s, with a wait interval of 25 s; 2000 pulses/day). The stimulation of the two hemispheres was performed sequentially at an interval of ten minutes. Each rTMS session was delivered 5 times/week on separate days for 4 weeks.

#### 5.2.3. MMPs Plasma Dosage

Whole blood samples were obtained by venous puncture at each of the three timepoints (T0, T1, and T2). The sampling was carried out using a test tube with anticoagulant (i.e., sodium citrate) to obtain the plasma after centrifugation. Plasma samples were stored frozen at −80 °C. Commercially available ELISA kits were used to determine the concentration of MMP1, -2, -9, -and -10 and of the respective inhibitors (TIMP1 and TIMP2) in sodium citrate–plasma. Assays were performed by following the instructions of the manufacturer (Merck, Darmstadt, Germany). The samples were analyzed with an ELISA reader, which reads the absorbance of the analyte by irradiating it at a certain wavelength (450 nm). The concentration measurement is obtained starting from the absorbance value. All standards and samples were assayed in duplicate. We used the following dilution: 1:10 for MMP1, 1:5 for MMP2, 1:600 for MMP9, 1:6 for MMP10, 1:25 for TIMP1, and 1:300 for TIMP2 ELISA.

### 5.3. Statistical Analysis

At baseline, we used the Mann–Whitney test (U) or Pearson’s chi-squared test (χ2) to compare the MCI-TMS and MCI-C on demographics (i.e., age, education, and sex) and cognitive (i.e., RBANS subtests) and behavioral (i.e., BDI-II, BAI, and AES) measures. To test the effects of TMS on cognitive and behavioral functioning, we compared the MCI-TMS and MCI-C on RBANS and behavioral scores at T1 and T2 by Quade’s rank analyses of covariance, using the baseline measures as covariates [76]. All multiple comparisons were corrected according to the Benjamini–Hochberg procedure, in which a corrected *p*-value lower than 0.05 was considered statistically significant. All analyses were performed using the IBM Statistical Package for Social Science (SPSS) version 25 (Chicago, IL, USA). MMPs/TIMPs’ plasmatic concentrations were expressed in Log_10_ scale. Multivariate tests of significance were performed to compare MMP1, -2, -9, and -10 and TIMP1 and TIMP2 levels in different time points. The Mann–Whitney test was used to compare results obtained between the two groups of subjects. A regression analysis (Pearson’s linear correlation) was applied for the correlation of plasmatic levels with clinical variables. Data were analyzed using SigmaPlot version 10.0 software and expressed as mean ± standard deviation (SD), with *p* ≤ 0.05 considered significant, using the Bonferroni method for multiple comparisons.

## Figures and Tables

**Table 1:** Demographics and cognitive and behavioral measures at baseline.

Variable	MCI-TMS (*n* = 10)	MCI-C (*n* = 10)	U-Test	*p*-Value	Adj-*p*
***Demographics***					
Age (y)	66.50 (62.25, 74,25)	70.50 (62.00, 75.00)	43.00	0.596	0.715
Education (y)	13.00 (9.50, 13.00)	10.50 (8.00, 13.00)	41.00	0.480	0.715
Gender (M)	4 (40.00%)	4 (40.00%)	0.00	1.000	1.000
***Cognitive assessment—RBANS subtests***					
List Learning	20.00 (15.75, 22.50)	17.00 (14.00, 22.25)	39.50	0.425	0.715
Story Memory-IR	15.00 (13.50, 17.00)	12.50 (9.75, 16.25)	32.50	0.182	0.546
Figure Copy	15.50 (12.00, 17.25)	12.00 (10.00, 15.00)	21.50	0.030	0.540
Line Orientation	14.50 (11.75, 17.25)	13.50 (10.25, 17.25)	42.00	0.544	0.715
Picture Naming	10.00 (10.00, 10.00)	9.50 (8.75, 10.00)	32.00	0.092	0.546
Semantic Fluency	13.00 (9.75, 15.25)	15.50 (12.50, 17.00)	30.50	0.137	0.546
Digit Span	6.00 (5.75, 8.50)	7.00 (6.00, 10.00)	39.50	0.409	0.715
Coding	27.00 (16.00, 34.75)	19.50 (13.75, 31.75)	35.50	0.272	0.699
List Recall	0.50 (0.00, 3.75)	1.00 (0.00, 3.50)	50.00	1.000	1.000
List Recognition	13.00 (12.25, 17.25)	15.50 (15.00, 17.00)	32.00	0.169	0.546
Story Recall-DR	0.50 (0.00, 7.25)	5.50 (3.75, 7.00)	31.00	0.145	0.546
Figure Recall	3.00 (0.00, 7.25)	3.00 (2.00, 7.75)	42.00	0.542	0.715
***Behavioral*** ***measures***					
BDI-II	15.50 (2.50, 22.00)	12.00 (8.00, 21.75)	49.50	0.970	1.000
BAI	4.00 (2.75, 16.50)	8.50 (2.75, 13.75)	42.50	0.569	0.715
AES	39.00 (32.00, 42.50)	39.00 (30.25, 40.00)	42.50	0.569	0.715

Table legend: MCI-TMS, patients with mild cognitive impairment who underwent TMS; MCI-C, patients with mild cognitive impairment who underwent sham TMS; Adj-*p*, *p*-value corrected for multiple comparisons (Benjamini–Hochberg procedure); y, years; M, male; Story Memory-IR or -DR, story memory immediate/delayed recall; BDI-II, Beck Depression Inventory II scale; BAI, Beck Anxiety Inventory; AES, Apathy Evaluation Scale. Between-group comparison, Mann–Whitney U-test. Data reported as median (25th, 75th percentile) or count (percentage).

**Table 2:** Cognitive and behavioral measures at T1 (4 weeks after rTMS).

Variable	MCI-TMS (*n* = 10)	MCI-C (*n* = 10)	*Quade*-Test	*p*-Value	Adj-*p*
***Cognitive assessment—RBANS subtests***					
List Learning	22.00 (18.50, 28.00)	18.00 (15.50, 23.25)	1.39	0.254	0.960
Story Memory-IR	14.00 (9.00, 17.00)	11.50 (5.50, 16.25)	0.05	0.813	0.983
Figure Copy	14.50 (11.50, 16.25)	11.00 (9.50, 12.25)	1.37	0.256	0.960
Line Orientation	15.00 (13.75, 16.25)	12.50 (9.25, 16.00)	1.80	0.196	0.960
Picture Naming	10.00 (9.00, 10.00)	10.00 (9.00, 10.00)	0.31	0.582	0.983
Semantic Fluency	9.00 (5.75, 13.25)	8.00 (5.75, 9.00)	1.03	0.323	0.969
Digit Span	8.00 (6.00, 10.00)	9.00 (5.75, 10.50)	0.00	0.983	0.983
Coding	29.50 (21.25, 36.75)	25.50 (14.50, 38.50)	0.08	0.770	0.983
List Recall	0.50 (0.00, 3.50)	1.00 (0.00, 3.25)	0.00	0.954	0.983
List Recognition	16.00 (13.00, 17.00)	15.00 (13.00, 18.25)	0.29	0.592	0.983
Story Recall-DR	2.00 (0.00, 7.25)	6.00 (3.75, 7.00)	0.12	0.734	0.983
Figure Recall	1.50 (0.00, 10.25)	4.00 (2.75, 9.25)	2.91	0.105	0.960
***Behavioral measures***					
BDI-II	9.00 (3.25, 17.25)	7.75 (8.50, 8.50)	0.17	0.683	0.983
BAI	4.00 (1.75, 26.00)	3.50 (2.75, 16.25)	0.02	0.884	0.983
AES	36.50 (34.75, 41.75)	34.50 (28.00, 40.25)	0.08	0.776	0.983

Table legend: MCI-TMS, patients with mild cognitive impairment who underwent TMS; MCI-C, patients with mild cognitive impairment who underwent sham TMS; Adj-*p*, *p*-value corrected for multiple comparisons (Benjamini–Hochberg procedure). Story Memory-IR or -DR, story memory immediate/delayed recall; BDI-II, Beck Depression Inventory II scale; BAI, Beck Anxiety Inventory; AES, Apathy Evaluation Scale. Between-group comparison, Quade’s rank analysis (baseline scores as covariate). Data reported as median (25th, 75th percentile) or count (percentage).

**Table 3:** Cognitive and behavioral measures at T2 (24 weeks after rTMS).

Variable	MCI-TMS (*n* = 10)	MCI-C (*n* = 10)	*Quade*-Test	*p*-Value	Adj-*p*
***Cognitive assessment—RBANS subtests***					
List Learning	19.50 (17.50, 21.25)	20.50 (16.00, 24.00)	1.58	0.224	0.586
Story Memory-IR	12.00 (6.50, 15.00)	9.00 (7.50, 13.50)	0.50	0.486	0.608
Figure Copy	14.50 (12.75, 15.25)	11.50 (9.75, 15.00)	0.52	0.477	0.608
Line Orientation	16.50 (12.75, 18.25)	12.50 (9.00, 14.25)	12.26	0.002	0.038
Picture Naming	8.00 (7.50, 9.50)	7.50 (7.00, 8.50)	0.02	0.879	0.878
Semantic Fluency	7.50 (6.00, 9.00)	8.00 (7.00, 9.25)	0.56	0.463	0.608
Digit Span	8.00 (5.75, 10.00)	7.00 (5.75, 9.25)	1.07	0.313	0.586
Coding	24.00 (21.00, 32.50)	30.50 (28.75, 34.25)	8.69	0.008	0.064
List Recall	0.00 (0.00, 1.00)	2.00 (0.75, 3.25)	6.70	0.018	0.092
List Recognition	12.50 (11.75, 15.50)	16.00 (14.50, 18.50)	4.89	0.040	0.150
Story Recall-DR	1.00 (0.00, 7.00)	5.50 (2.50, 6.25)	0.13	0.722	0.777
Figure Recall	2.00 (0.00, 7.25)	4.00 (3.00, 7.00)	1.39	0.253	0.586
***Behavioral measures***					
BDI-II	12.00 (5.00, 26.50)	8.00 (7.00, 18.50)	0.12	0.726	0.777
BAI	5.00 (3.00, 19.00)	7.00 (4.50, 14.00)	0.89	0.357	0.595
AES	33.00 (27.00, 46.50)	37.00 (31.00, 46.50)	1.08	0.312	0.586

Table legend: MCI-TMS, patients with mild cognitive impairment who underwent TMS; MCI-C, patients with mild cognitive impairment who underwent sham TMS; Adj-*p*, *p*-value corrected for multiple comparisons (Benjamini–Hochberg procedure). Story Memory-IR or -DR, story memory immediate/delayed recall; BDI-II, Beck Depression Inventory II scale; BAI, Beck Anxiety Inventory; AES, Apathy Evaluation Scale. Between-group comparison, Quade’s rank analysis (baseline scores as covariate). Data reported as median (25th, 75th percentile) or count (percentage). Statistically significant differences are shown in **bold**.

## Data Availability

All data and materials support the reported claims and comply with standards of data transparency. Data will be made available upon reasonable request.
